# Epigenetic therapy in urologic cancers: an update on clinical trials

**DOI:** 10.18632/oncotarget.14226

**Published:** 2016-12-26

**Authors:** Inês Faleiro, Ricardo Leão, Alexandra Binnie, Ramon Andrade de Mello, Ana-Teresa Maia, Pedro Castelo-Branco

**Affiliations:** ^1^ Regenerative Medicine Program, Department of Biomedical Sciences and Medicine, University of Algarve, Faro, Portugal; ^2^ Centre for Biomedical Research, University of Algarve, Faro, Portugal; ^3^ Algarve Biomedical Center, Campus Gambelas, Edificio 2. Faro, Portugal; ^4^ Department of Surgery, Princess Margaret Cancer Center, Division of Urology, University of Toronto, Toronto, Canada; ^5^ Renal Transplantation and Urology Service, Coimbra University Hospital Center EPE, Faculty of Medicine, University of Coimbra, Portugal

**Keywords:** epigenetic therapy, urologic cancers, clinical trials

## Abstract

Epigenetic dysregulation is one of many factors that contribute to cancer development and progression. Numerous epigenetic alterations have been identified in urologic cancers including histone modifications, DNA methylation changes, and microRNA expression. Since these changes are reversible, efforts are being made to develop epigenetic drugs that restore the normal epigenetic patterns of cells, and many clinical trials are already underway to test their clinical potential. In this review we analyze multiple clinical trials (*n*=51) that test the efficacy of these drugs in patients with urologic cancers. The most frequently used epigenetic drugs were histone deacetylase inhibitors followed by antisense oligonucleotides, DNA methyltransferase inhibitors and histone demethylase inhibitors, the last of which are only being tested in prostate cancer. In more than 50% of the clinical trials considered, epigenetic drugs were used as part of combination therapy, which achieved the best results. The epigenetic regulation of some cancers is still matter of research but will undoubtedly open a window to new therapeutic approaches in the era of personalized medicine. The future of therapy for urological malignancies is likely to include multidrug regimens in which epigenetic modifying drugs will play an important role.

## INTRODUCTION

Urologic cancers account for approximately 10% of all cancer deaths in the USA and include bladder, kidney, prostate and testicular cancers [1].

The establishment and progression of malignancy involves broad changes in gene expression that are determined by both genetic and epigenetic events. Genetic events include chromosome rearrangements and duplications as well as translocations, deletions, and single base pair mutations. Epigenetic modifications are somatically heritable changes that modify gene expression without altering the DNA sequence. Among these are histone modifications, DNA methylation, and miRNA expression [2].

## HISTONE MODIFICATIONS

Post-translational modification of the histone protein N terminal tails can alter the structure of the nucleosome and change the compaction state of chromatin. Common modifications include methylation, acetylation, phosphorylation, ubiquitylation and sumoylation [2]. Among these, histone acetylation and methylation are best described in cancer epigenetic dysregulation [2].

Histone acetylation neutralizes the positive charge of lysine residues, weakening their electrostatic interactions with DNA [3]. This leads to a more relaxed state of the chromatin and is associated with transcriptional activation. Addition of the acetyl group is carried out by histone acetyltransferases (HATs), and its removal is catalyzed by histone deacetylases (HDACs) [4]. HDACs are classified into four distinct groups based on their homology to yeast histone deacetylases. Class I HDACs encompass HDAC1, 2, 3 and 8 and, with the exception of HDAC8 that can be located in the nucleus or cytoplasm, are exclusively located in the nucleus. Class II HDACs include HDAC4, 5, 6, 7, 9 and 10 and can be present in the nucleus or the cytoplasm [5, 6]. Class III HDACs are the sirtuins, proteins which require the cofactor NAD+ to be active. Unlike Class I and Class II HDACs, sirtuins are not inhibited by known pharmacologic HDAC inhibitors (HDACi) such as Vorinostat and Trichostatin A (TSA) [5, 6].

Histone methylation occurs at lysine and arginine residues and, depending on the target, can lead to activation or repression of gene expression [7]. Methylation is catalyzed by histone methyltransferases (HTMs) while demethylation is performed by histone demethylases (HDMs). There are currently two histone demethylase families, the lysine specific demethylases (LSD) and the JmjC-domain-containing histone demethylases (JHDMs) [7]. The LSDs comprise LSD1 and LSD2, which are dependent on FAD to be catalytically active [8]. The JHDMs in turn, catalyze the hydroxylation of the lysine methylgroup and require two factors to be catalytically active: Fe(II) and 2-oxoglutarate [7].

In cancer, histone modifications have been associated with both activation and repression of gene expression. Modifications such as histone 3 methylation at lysine 4 (H3K4me), histone 3 di-methylation at lysine 4 (H3K4me2), histone 3 tri-methylation at lysine 4 (H3K4me3), histone 3 acetylation at lysine 9 (H3K9ac), histone 3 methylation at lysine 9 (H3K9me) and histone 3 acetylation at lysine 27 (H3K27ac) are associated with active chromatin whereas histone 3 tri-methylation at lysine 36 (H3K36me3), histone 3 tri-methylation at lysine 9 (H3K9me3) and histone 3 methylation at lysine 27 (H3K27me) are associated with repressive chromatin [9].

## DNA METHYLATION

DNA methylation results from addition of a methyl group to the 5-carbon of a cytosine residue by the enzyme DNA methyltransferase (DNMT) [10]. DNMT forms a complex with CpG dinucleotides that allows the transfer of a methyl group to the cytosine residue [10]. Many CpG sites are located in the promoter regions of genes. Collectively they are known as CpG islands. In general, DNA methylation of CpG islands located in gene promoters leads to transcriptional repression. However, there are exceptions to this classical view, in which promoter hypermethylation is associated with increased gene expression [11–14]. This occurs in instances where DNA methylation drives the use of an alternative transcription start site or inhibits the binding of a repressive protein [15]. Increased gene expression in the context of promoter hypermethylation is associated with an increase in H3K4me3, a histone mark characteristic of gene activation [15].

In eukaryotes, DNA methylation is mediated by three DNMTs: DNMT1 is responsible for the maintenance of methylation patterns after DNA replication whereas DNMT3A and DNMT3B carry out *de novo* methylation [4]. Any alteration that affects the activity of these enzymes can lead to an imbalance in methylation that provides the basis, or contributes, to the initiation of carcinogenesis.

## MIRNAS

miRNAs are small endogenous non-coding RNAs (ncRNAs), 21-25 nucleotides in length, that regulate gene expression by targeting specific messenger RNAs (mRNAs) for translational repression or degradation. Expression patterns of miRNAs differ between normal and tumor tissues [16, 17]. Depending on their target, miRNAs can act either as tumor suppressors or oncogenes; downregulation of an miRNA that targets an oncogene, or an overexpression of an miRNA that targets a tumor suppressor gene, can promote carcinogenesis [16, 17].

## EPIGENETIC DRUGS

Two strategies for epigenetic therapy are currently in use: small molecules that inhibit epigenetic-modifying enzymes and manipulation of miRNA expression.

Amongst the small molecule inhibitors are HDAC inhibitors and DNMT inhibitors. HDAC inhibitors (HDACi) are classified into 4 groups according to their chemical structures: hydroxamates (SB393, Vorinostat, Panobinostat), cyclic peptides (Romidepsin), benzamides (Entinostat and Mocetinostat) and aliphatic fatty acids (Valproic Acid) [18].

The majority of HDACi inhibit zinc-dependent HDACs by interacting with the zinc ion. In cancer cells, the inhibition of histone deacetylation restores expression of tumor suppressor genes that were previously silenced by epigenetic mechanisms [18, 19].

DNMT inhibitors are divided into nucleoside analogues and non-nucleoside analogs [4]. Nucleoside analogues, such as Azacitidine, Decitabine and FdCyd, are cytosine analogs modified at the C5 position. Inside the cell they are metabolized and incorporated into DNA molecules [4]. DNA methyltransferases can bind to these modified nucleotides but their modification at C5 prevents their methylation. It also prevents the dissociation of the enzyme thereby reducing DNMT activity at other sites [4]. Non-nucleoside analogues, such as Hydralazine, Procainamide and MG98, inhibit methylation by binding to the catalytic region of the enzyme [4].

Another focus of epigenetic therapy is the manipulation of miRNA expression and activity. Several strategies have been employed to silence miRNAs that are overexpressed in cancer. These include anti-miRNA oligonucleotides (AMOs), peptide nucleic acids (PNAS), miRNA-masking antisense oligonucleotides (miR-mask) and miRNA sponges [16]. Restoration of miRNA expression that has been downregulated in cancer is achieved by administration of synthetic miRNAs or by induced expression of miRNA coding genes using viral constructs, such as adenovirus-associated vectors [16].

**Figure 1:**
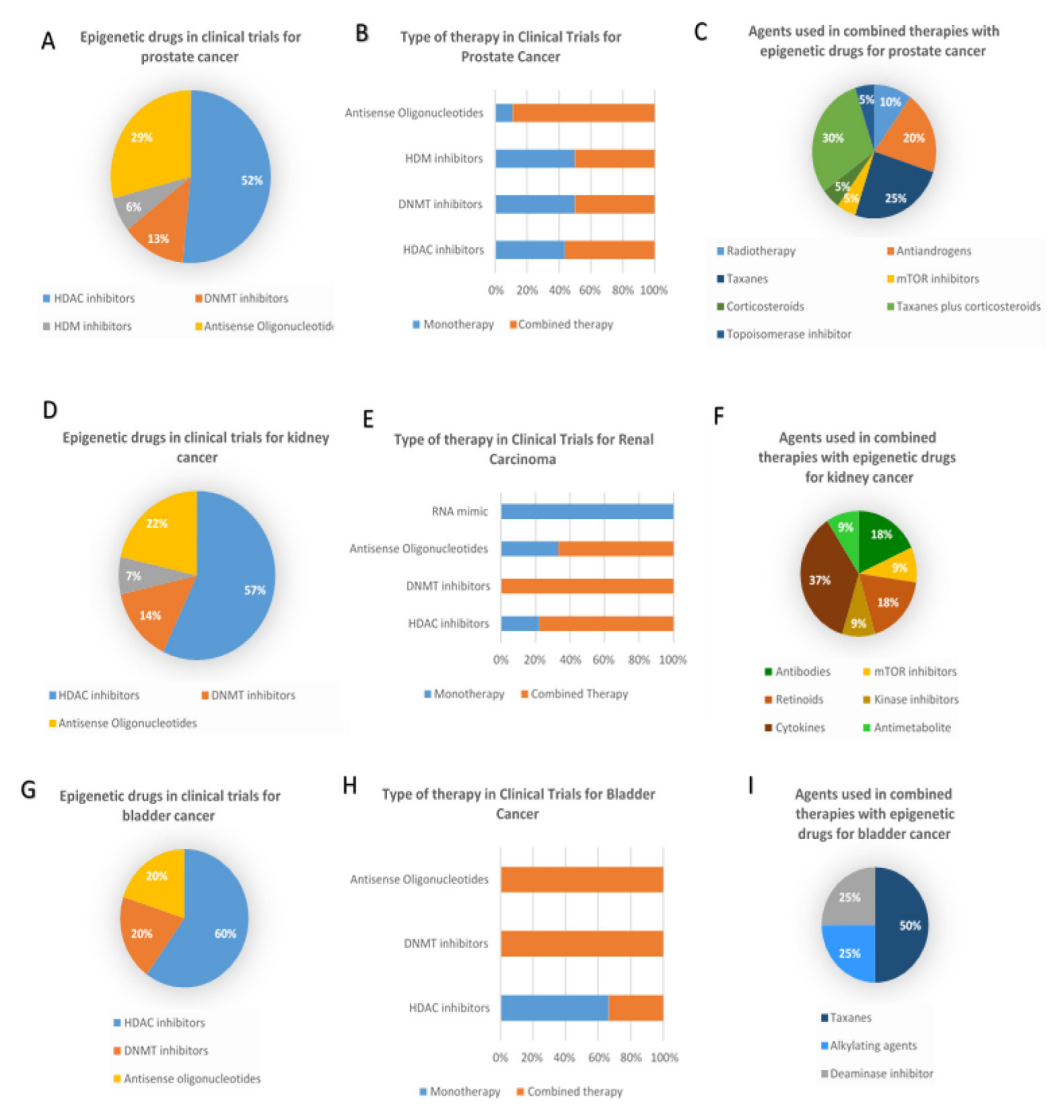
Epigenetic therapies in clinical trials for prostate, bladder and kidney cancers **A**. Percentage of clinical trials employing each types of epigenetic therapeutic agents in prostate cancer; **B**. Percentage of clinical trials using mono or combined therapy as therapeutic strategy with the different classes of epigenetic drugs in prostate cancer; **C**. Percentage of clinical trials where different agents are used in combined therapies for prostate cancer; **D**. Percentage of clinical trials employing each types of epigenetic therapeutic agents in kidney cancer; **E**. Percentage of clinical trials using mono or combined therapy as therapeutic strategy with the different classes of epigenetic drugs in kidney cancer; **F**. Percentage of clinical trials where different agents are used in combined therapies for kidney cancer **G**. Percentage of clinical trials employing each types of epigenetic therapeutic agents in bladder cancer; **H**. Percentage of clinical trials using mono or combined therapy as therapeutic strategy with the different classes of epigenetic drugs in bladder cancer; **I**. Percentage of clinical trials where different agents are used in combined therapies for bladder cancer

Dysregulation of epigenetic marks leads to changes in gene expression that, in cancer cells, can result in activation of oncogenes or inactivation of tumor suppressor genes, both of which can contribute to cancer. Unlike genetic mutations, however, epigenetic changes are reversible. Therefore, the development of drugs capable of restoring the normal epigenetic patterns of cells has great therapeutic potential. In this review we discuss the efficacy of this novel therapeutic approach through the analysis of clinical trials of epigenetic therapies conducted in prostate, kidney and bladder cancers.

## METHODS

We performed a comprehensive literature review and searched for clinical trials from the United States (https://clinicaltrials.gov/) and European (https://www.clinicaltrialsregister.eu/) databases. Relevant articles on the subject were also retrieved from PubMed database using keywords encapsulating all types of epigenetic therapies and urologic cancers (examples: “epigenetic therapy” AND “urologic cancer”, “prostate cancer” AND “HDACi”, “kidney cancer” AND “DNMTi”). To guarantee that most of the data on the subject was included, the reference sections of the captured articles were also filtered for relevant articles.

### Prostate cancer - epigenetics

Dysregulation of epigenetic-modifying enzymes disturbs normal epigenetic patterns and is associated with cancer development and progression. In prostate cancer, DNA methyltransferases are upregulated [20, 21]. Histone-modifying enzymes, such as HDACs are upregulated in prostate cancer [22]. HMTs and HDMs show variable changes in expression with a tendency for upregulation of HMTs and lower expression of HDMs [23, 24]. Prognostically, overexpression of HDAC2 is associated with a shortened time before prostate cancer recurrence as shown in a subgroup of patients with Gleason Score 7 carcinomas, [6].

Specific histone modifications have also been associated with prostate cancer [25, 26]. The levels of histone marks H3Ac and H3K9me2 are significantly lower in tumor tissue when compared to normal tissue [26]. Conversely, an increase in H3K27me3 is found in metastatic tissue relative to localized tumors and normal prostatic tissue [25]. Finally, higher levels of H3K4me1 are associated with a higher probability of recurrence [26].

Changes in DNA methylation are also evident in prostate cancer and are targets for epigenetic therapy. The *CCDN2*, *GSTP1* and *RARβ2* genes, involved in cell cycle control, DNA repair mechanisms and hormonal responses respectively, are hypermethylated in prostate cancer. Alteration of their normal methylation status is correlated with poor clinical prognosis [26]. As with many epigenetic alterations, these biomarkers are useful in diagnosis and prognosis of disease [25, 26].

Finally, miRNA levels are also altered in prostate cancer, affecting the expression of genes involved in cell cycle control, apoptosis, migration, and invasion [27]. Levels of miRNAs also have the potential to be used as biomarkers for diagnosis and prognosis [25]. As an example, miR-141 is upregulated in prostate cancer [25, 28]. Serum levels of this miRNA can distinguish between tumor and healthy tissue and higher levels of miR-141 are associated with worse prognosis [28]. miR-449a is another miRNA that is downregulated in prostate cancer. It targets HDAC1, so its downregulation contributes to overexpression of this enzyme, showing that epigenetic-modifying enzymes are often regulated epigenetically [27, 29].

### Prostate cancer - current treatment

Prostate cancer treatment is disease stage-specific. Epigenetic therapies have thus far been limited to the advanced form of castrate resistant prostate cancer (CRPC). Currently there are several chemotherapeutic agents approved for the treatment of advanced CRPC: Sipuleucel T, Docetaxel, Cabazitaxel, Abiraterone, Alpharadin and Enzalutamide [30–35]. Although all of these agents have shown efficacy, strategies for the sequence of administration and their combination are still being optimized [36]. Treatment resistance is a major concern with some of these agents, including Abiraterone and Enzalutamide, reflecting the need for ongoing development of novel therapeutic strategies [36]. Since epigenetic dysregulation contributes to the development of treatment resistance, epigenetic therapy is an intriguing addition to the CRPC therapy arsenal [37].

### Prostate cancer - pre-clinical data

Pre-clinical studies in prostate cancer cell lines demonstrate that treatment with HDACi can restore susceptibility to chemotherapeutic agents such as taxanes, antiandrogens, and mTOR inhibitors [38–40]. Combined therapy using HDACi and taxanes prevents tumor growth and increases cell death rate when compared to a monotherapeutic approach [38]. Liu *et al* showed that low doses of the HDACi Panobinostat can restore the susceptibility of prostate cancer cells to hormonal therapy with the nonsteroidal antiandrogen Bicalutamide [39]. The HDACi Belinostat (PXD101) can also downregulate the androgen receptor, preventing the onset of castration resistant prostate cancer *in vivo* in the context of hormonal therapy [41].

An *in vitro* study of the HDACi Panobinostat in combination with the mTOR inhibitor Rapamycin in prostate cancer cell lines resulted in a decrease in *HIF1- α* expression leading to inhibition of angiogenesis [40]. Combined therapy with these agents was more efficient than either one administrated alone [40].

Cancer stem cells are thought to be responsible for treatment resistance and tumor recurrence [42] and present epigenetic alterations that contribute to their ability to resist therapy [42]. Epigenetic therapeutics may therefore have the potential to target not only the bulk tumor but also this key subset of cells [42]. A study carried out by Frame *et al* revealed that prostate stem-like cells are more resistant to radiotherapy [43]. However, combined therapy with HDACi restored sensibility to radiotherapy [43]. Additionally, prostate stem-like cells treated jointly with the HDACi Trichostatin A and radiotherapy showed a significant reduction in the number of cell colonies formed when compared to treatment with radiation alone [43].

Cancer is a heterogeneous disease and the identification of biomarkers that predict whether a specific therapy (including epigenetic therapies) will be beneficial, is essential to improving cancer treatment. Recently, it has been reported that prostate cells positive for the presence of androgen receptor and cellular prostatic acid phosphatase show greater response to treatment with HDACi than cells without this pattern of expression [44].

At the level of DNA methylation, reversion of methylation can restore expression of genes silenced by this epigenetic mechanism. Treatment of human prostate cancer cells with Procainamide, a non-nucleoside DNMT inhibitor, results in a decrease in *GSTP1* methylation levels and a consequent increase in gene expression [45]. *In vivo*, treatment of immunodeficient mice carrying xenograft tumors with Procainamide resulted in a significant reduction in tumor size, suggesting clinical efficacy [45].

Resistance to hormonal therapy in prostate cancer is mediated by several mechanisms. Alterations at the DNA level include androgen receptor gene amplifications and point mutations [46]. However, these modifications account for only a minority of cases. Downstream activation of the androgen receptor pathway and activation of an alternative signaling pathway can also contribute to hormonal therapy resistance [46]. Hypermethylation of the androgen receptor promoter region correlates with decreased androgen receptor expression and is also associated with the development of hormonal therapy resistance [47]. *In vivo* studies reveal that long-term treatment of prostate cancer cells with the DNMTi Azacitidine led to a significant reduction in cell proliferation due to increased androgen receptor expression. Moreover, androgen receptor induction restored sensitivity to the antiandrogen agent Bicalutamide [47].

The DNA demethylating agent, Disulfiram, has also been tested in prostate cancer cells. Treatment with Disulfiram resulted in the reestablishment of *APC* and *RARβ* gene expression, both of which are known to be hypermethylated and inactive in prostate cancer [48]. Cell growth inhibition was observed *in vitro,* and *in vivo* using prostate cancer xenograft models [48].

Finally, microRNAs modulators have been tested in preclinical studies as potential therapeutic options for prostate cancer. miR-16 regulates the expression of genes involved in cell-cycle control and apoptosis such as *CDK1*, *CDK2* and *BCL2* [49, 50]. Transfection of a synthetic miR-16 reduced the proliferative capacity of several prostate cancer cell lines [49]. *In vivo*, Takeshita *et al* used the atelocollagen method to deliver miR-16 to bone metastases via the mouse tail vein. They subsequently observed a suppression in metastasis growth, indicating not only efficacy of the treatment but also of the delivery method [49].

Like HDACi and DNMTi, antisense oligonucleotides can restore the sensitivity of cancer cells to chemotherapeutic agents. Upregulation of the *Bcl2* and *CLU* genes in prostate cancer is linked to chemoresistance and cancer progression [51, 52]. Knockdown of these genes by antisense oligonucleotides decreases gene expression and reestablishes tumor sensitivity to taxane-based chemotherapy [51, 52]. Furthermore, transfection of miR-449a into prostate cancer cells lines caused cell cycle arrest and a decrease in HDAC1 levels, an effect also observed after knockdown of *HDAC1* using an siRNA [29]. The inhibitory effect of miR-449a on cell cycle progression was associated with increased expression of the protein p27 [29].

These results demonstrate the potential for epigenetic therapies to advance prostate cancer treatment.

### Prostate cancer - clinical data

Clinical trials using HDAC inhibitors for the treatment of prostate cancer showed a PSA response in five studies, three of which resulted in a decrease in PSA levels of ≥50% (Table 1: NCT01075308, NCT00667862, NCT00330161, NCT00106418, [53–57]). The best results were obtain with the administration of the HDACi Panobinostat (Table 1: NCT00667862, [54, 55]. Stable disease was reported in two clinical trials, but in one of them conversion from an unfavorable circulating tumor cell profile to a favorable one was observed in 64% of the patients (Table 1: NCT01075308, NCT00667862, NCT00330161, [53, 54, 56]).

**Table 1:** Clinical trials of epigenetic drugs in prostate cancer

Drug	Combined Therapy	Enzimatic Class	Approval Stage	Status	Indication	Results	Reference/Clinical trial identification
SB939	-	HDAC inhibitor	Phase 2	Completed	Castration Resistance Prostate Cancer (CRPC)	6% of the patients had a PSA response64% of the patients had a conversion from an unfavorable CTC profile to a favorable one	Eigl *et al*. 2015 (NCT01075308)
Panobinostat	-	HDAC inhibitor	Phase 2	Completed	CRPC	14,3% of the patients had a PSA decrease <50% but no objective responses were seen 11,4% of the patients had stable disease for at least 24 weeks	Rathkopf *et al*. 2013 (NCT00667862)
Panobinostat	Docetaxel	HDAC inhibitor	Phase 1	Completed	CRPC	63% had a PSA decrease >= 50%	Rathkopf *et al*. 2010
Panobinostat	Radiotherapy	HDAC inhibitor	Phase 1	Completed	Prostate Cancer, esophageal cancer and neck cancer	No study results or publications provided	NCT00670553
Panobinostat	Docetaxel/prednisone	HDAC inhibitor	Phase 2	Completed	CRPC	No study results or publications provided	NCT00878436
Panobinostat	Bicalutamide	HDAC inhibitor	Phase 1	Completed	CRPC	No study results or publications provided	NCT00663832
Vorinostat	-	HDAC inhibitor	Phase 2	Completed	Progressive metastatic prostate cancer	No PSA declines >=50% were observed Median of progression free survival=2,8 months with a median overall survival of 11,7 months	Bradley *et al*. 2010 (NCT00330161)
Vorinostat	Docetaxel	HDAC inhibitor	Phase 1	Terminated	Advanced solid tumor including prostate cancer, urothelial carcinoma and kidney cancer	This study was terminated due to excessive toxicity as five patients experienced dose-limiting toxicities (DLT) No responses were observed	Schneider *et al*. 2012 (NCT00565227)
Vorinostat	Temsirolimus	HDAC inhibitor	Phase 1	Terminated	Metastatic prostate cancer	This study was terminated due to lack of efficacy	NCT01174199
Vorinostat	Androgen deprivation therapy	HDAC inhibitor	Phase 2	Completed	Localized prostate cancer	No study results or publications provided	NCT00589472
Vorinostat	-	HDAC inhibitor	Phase 1	Completed	Advanced solid tumors including prostate cancer	No study results or publications provided	NCT00005634
Romidepsin	-	HDAC inhibitor	Phase 2	Completed	Metastatic prostate cancer	No study results or publications provided	NCT00106418
Romidepsin	-	HDAC inhibitor	Phase 2	Completed	metastatic castration-resistant prostate cancer (MCRPC)	63% of the patients had progressive disease with a median time to progression of 49,5 daysPSA decline >=50% was observed in 5,7% of the patients	Molife *et al*. 2009
Curcumin	Docetaxel	HDAC inhibitor	Phase 2	Ongoing	MCRPC	Final data collection date for primary outcome measure: January 2017	NCT02095717
Curcumin	-	HDAC inhibitor	Phase 2	Ongoing	Prostate cancer	Estimated primary completion date: June 2020	NCT02064673
Curcumin	Radiotherapy	HDAC inhibitor	-	Completed	Prostate cancer	No PSA response was observed but the severity of radiotherapy related urinary symptoms was reduced,	Hejazi J. *et al*. 2013
Dissulfiram	-	DNMT inhibitor	Phase 1	Completed	Non-metastatic recurrent prostate cancer	Five patients achieve a transient demethylation response Six patients discontinue therapy due to adverse effects	Schweizer *et al*. 2013
Azacitidine	Combined Androgen Blockade (CAB)	DNMT inhibitor	Phase 2	Completed	CRPC	Overall median PSA doubling time increased significantly (2.8 vs 1.5 months of the baseline).Median of progression free survival=12,4 weeksFourteen patients had some PSA decline and 1 patient had a PSA decline >=30%	Sonpavde *et al*. 2011
Azacitidine	-	DNMT inhibitor	Phase 2	Completed	Prostate cancer	No study results or publications provided	NCT00384839
Azacitidine	Docetaxel/prednisone	DNMT inhibitor	Phase1/2	Terminated	CRPC	This study was terminated due to withdrawal of funding Complete and partial response were achieved by one and two patients, respectively	NCT00503984

**Table:** 

Phenelzine sulfate	-	HDM inhibitor/monoamine oxidase A inhibitor	Phase 2	Ongoing	Non-metastatic recurrent prostate cancer	Study completion date: August 2018	NCT02217709
Phenelzine sulfate	Docetaxel	HDM inhibitor/monoamine oxidase A inhibitor	Phase 2	Ongoing	Progressive prostate cancer	Final data collection date for primary outcome measure: January 2016	NCT01253642
OGX-011	Docetaxel/prednisone	Antisense oligonucleotide that targets clusterin	Phase 1	Completed	advanced cancer including prostate, bladder and kidney cancer	Six patients with hormone-refractory prostate cancer had a PSA decline >=50%	Saad et al. 2011 (NCT00471432)
OGX-011	Docetaxel/prednisone and docetaxel/mitoxantrone	Antisense oligonucleotide that targets clusterin	Phase 3	Completed	MCRPC	No objective responses were seen 41% of the patients discontinued treatment due to serious adverse events	Chi et al. 2008 (NCT01188187)
OGX-011	Cabazitaxel/prednisone	Antisense oligonucleotide that targets clusterin	Phase 3	Ongoing	CRPC	Study completion date: December 2016	NCT01578655
Oblimersen	Docetaxel	Antisense oligonucleotide that targets Bcl-2	Phase 2	Completed	CRPC	PSA response was observed in 46% and 37% of the patients treated with docetaxel alone and docetaxel+oblimersen, respectivelyPartial response was observed in 18% and 24% of the patients in the referred groups and major toxic events were reported in 22,8% and 40,7% respectively	Sternberq *et al*. 2009 (NCT00085228)
Oblimersen sodium (Genasense)	Mitoxantrone	Antisense oligonucleotide that targets Bcl-2	Phase 1	Completed	CRPC	Two patients had a PSA reduction >=50%, 1 patient had a PSA resuction <50%, and 5 patients had stable disease	Chi *et al*. 2001
OGX-427	Prednisone	Antisense oligonucleotide that targets heat shock protein27	Phase 2	Completed	CRPC	No study results or publications provided	NCT01120470
OGX-427	Abiraterone	Antisense oligonucleotide that targets heat shock protein27	Phase 2	Ongoing	MCRPC	Study completion date: December 2017	NCT01681433
ISIS 1837	Docetaxel/prednisone	Antisense oligonucleotide that targets eIF4E	Phase 2	Completed	Metastatic resistant castrate prostate cancer	No study results or publications provided	EudraCT Number: 2010-022239-12
ISIS 3521/ISIS 5132	-	Antisense oligonucleotides that targets PKC-α and Raf-1, respectively	Phase 2	Completed	CRPC	No objective responses were observed but three patients had stable disease for 5 or more months PSA values of five patients did not rise more than 25% for >=120 days	Tolcher *et al*. 2002
LY2181308	Docetaxel/prednisone	Antisense oligonucleotide that targets survivin	Phase 2	Completed	CRPC	No differences in efficacy were observed between the control and the experimental group. Higher incidence of adverse effects in the LY2181308 treated group.	Wiechno *et al*. 2014

Most common side effects were grade 2 fatigue and nausea. In addition, HDACi SB939 caused five patients to experience one or more grade 3 complications (Table 1: NCT01075308, [53]). More severe side effects were noted with HDACi Panobinostat, resulting in 71, 4% of the patients experiencing one or more grade 3 adverse effects and four subjects reporting grade 4 adverse effects (Table1: NCT00667862, [54]). Another trial using Panobinostat reported no grade 4 toxicities when administered as monotherapy [55], however, when administered in combination with Docetaxel, seven patients experienced grade 4 toxicities [55] (Table 1). HDACi Vorinostat also showed a complex side effect profile. When administered alone in patients pre-treated with chemotherapeutic agents it led to the development of grade 3/4 toxicities in 48% of the patients, with 41% of the patients forced to discontinue therapy due to their severity (Table 1: NCT00330161, [56]). A second trial of Vorinostat in combination with docetaxel was terminated early due to excessive toxicity as five patients experienced dose-limiting toxicities, including two patients experiencing neutropenic fever and sepsis. The other three patients reported an anaphylactic reaction, a myocardial infarction and a gastrointestinal bleed, respectively (Table 1: NCT00565227, [58]). Finally, a trial of the HDACi Romidepsin in metastatic prostate cancer resulted in no grade 4 toxicities, and grade 3 events represented only 4.7% of all reported adverse effects (Table 1: NCT00106418, [57]).

Curcumin, a compound found in the spice turmeric, is another HDACi [59]. A trial of Curcumin in prostate cancer showed no PSA response when used in combination with radiotherapy (Table 1: NCT01917890, [60]). However, there was a significant reduction in urinary symptoms, one of the most common side effects of radiotherapy (Table 1: NCT01917890, [60]). Two additional trials testing Curcumin in the treatment of prostate cancer are ongoing (Table 1: NCT02095717, NCT02064673).

DNMT inhibitors have also showed promising results in clinical trials of prostate cancer. When treated with the DNMTi disulfiram, five patients achieved a transient demethylation response. No grade 4 adverse effects were observed in this trial but 6 patients were forced to quit due to treatment toxicity (Table 1: NCT01118741, [61]). The DNMTi Azacitidine was trialed in chemonaive patients with CRPC in combination with combined androgen blockade (CAB). PSA doubling time increased relative to patients receiving only CAB and no grade 4 toxicities were reported, although 4 patients had to stop treatment due to grade 3 toxicities [62].

Azacitidine has also been tested in combination with Docetaxel and Prednisone in CRPC (Table 1: NCT00503984). Therapeutic response was assessed by magnetic resonance imaging with a complete response considered the disappearance of target lesions, and a partial response considered a ≥30% decrease in the sum of the longest diameter of targeted lesions. Complete and partial responses were achieved by only one and two patients, respectively. A PSA response was observed in 10 patients. Despite some positive results, the study was terminated due to withdrawal of funding (Table 1: NCT00503984). More studies are needed to assess the clinical potential of this agent.

Another category of epigenetic drugs with clinical potential in cancer treatment are HDM inhibitors. Phenelzine is a monoamine oxidase A (MAOA) inhibitor used in the treatment of psychiatric disease. MAOA is an enzyme responsible for the deamination of neurotransmitters, such as dopamine, serotonin and norepinephrine, that are important in a variety of neurological and psychiatric illnesses [63]. MAOA has close homology to LSD1, a histone demethylase, which catalyzes removal of the methyl group from H3K4me1 and H3K4me2. As a result of this homology, Phenelzine is able to bind and inhibit LSD1. [63]. Lower levels of H3K4me2 are correlated with higher risk of recurrence in prostate cancer [64]. Phenelzine is currently being trialed as a monotherapy for the treatment of recurrent prostate cancer and in combination with docetaxel for the treatment of progressive prostate cancer. Given that Phenelzine is already an approved medication, positive responses in these clinical trials will open the door to using this class of epigenetic drugs in clinical practice in the near future.

In the area of miRNA modulation, four clinical trials of antisense oligonucleotides have reported a positive PSA response, with three trials describing a PSA response >=50% (Table 1: NCT01188187, NCT00471432, NCT00085228, [65–68]). One of these trials, evaluating the efficacy of the antisense oligonucleotide OGX-011 in combination with docetaxel or mitoxantrone, reported a PSA decrease ≥50% in 6 of 14 patients with CRPC (Table 1: NCT00471432, [66]). Most of the adverse effects were grade 1 and 2 only, however adverse effects of grade 3 or higher affected 60% of the patients receiving the antisense oligonucleotide in combination with docetaxel/prednisone and 73% of the patients receiving the OGX-011 in combination with mitoxantrone/prednisone (Table 1: NCT00471432, [66]). The most common grade 3 or higher adverse effects in both groups were fatigue and lymphopenia (Table 1: NCT00471432, [66]). OGX-011 was also tested in combination with docetaxel and prednisone in a phase III clinical trial but, despite some positive results observed in phase II of the study where 58% of the patients had a PSA response >=50%, no significant results were observed in phase III (Table 1: NCT00471432, NCT01188187, [66, 69]). In the group receiving the combined therapy OGX-011/docetaxel/prednisone, 41% of the patients had to discontinue the treatment program due to adverse effects of the therapy (≥3 grade) (Table 1: NCT01188187, [69]).

Administration of the antisense oligonucleotide LY2181308 to decrease the expression of survivin, an anti-apoptotic gene involved in therapy resistance was tested in a randomized phase 2 clinical trial performed by Wiecho *et al* for the treatment of CRPC [70]. The patients allocated in the group treated with LY2181308 reported higher incidence of grade 3 and 4 adverse effects without any improvement in progression free survival or overall survival of the patients [70].

Disappointing results were obtained with another antisense oligonucleotide, Oblimersen, that was trailed by Sternberg *et al* both alone and in combination with docetaxel (Table 1: NCT00085228, [67]). The authors observed a PSA response in 46% of the patients treated with docetaxel alone, versus 37% of the patients treated with the antisense oligonucleotide and docetaxel (Table 1: NCT00085228, [67]). In the group of patients receiving the combined therapy, major toxic events were observed in 40.7% of the patients, compared to 22.8% of the patients receiving the antisense oligonucleotide alone, indicating that docetaxel increased oblimersen-related toxicity. This suggests that combined therapy with antisense oligonucleotides and taxanes might not the best therapeutic approach (Table 1: NCT00085228, [67]). In another clinical trial Oblimersen was administrated in combination with mitoxantrone, with 2 patients out of 25 showing a PSA decrease equal or superior to 50%. One patient had a PSA response inferior to 50% while stable disease was observed in a further five patients [68].

Although no clinical benefits were observed in a study testing the antisense oligonucleotides ISIS 3512 and ISIS 5132, two patients who received the oligonucleotide ISIS 3512 and one patient who received ISIS 5132 did not show disease progression for at least five months [71]. Finally, results are currently unavailable from eight completed clinical trials and two other trials are ongoing (Table 1).

### Kidney cancer - epigenetics

Genetic and epigenetic dysregulation of genes involved in pathways such as the hypoxia-inducible pathway, the mTOR pathway, and the *cMET-RAF-MEK-ERK* pathway contribute to the progression of kidney cancer [72]. Changes in the levels of epigenetic-modifying enzymes are an important factor in altering expression of genes involved in cancer-related pathways. In renal cell carcinoma (RCC), an increase in the levels of histone demethylases such as *UTX*, *JMJD2* and *EZH2*, results in a reduction in H3K27me and promotes progression of the disease [73, 74]. Also in RCC, almost 60% of patients overexpress *HDAC1* and *HDAC2* [75]. No prognostic value has been associated with these alterations [75]. *HDAC3* is also highly expressed, but only in the papillary carcinoma subset [75].

At the level of individual epigenetic changes, low levels of H3K4me2, H3K18Ac, and H3K9me2 are associated with poor prognosis and lower survival probability in RCC., Mechanistically, H3K4me2 and H3K18Ac are associated with active transcription while H3K9me2 is associated with transcriptional repression [73, 74]. H3K27me is another histone modification that correlates with poor clinical outcome, result of overexpression of histone demethylases in RCC [73, 74].

Clear cell RCC is the most common form of renal cell carcinoma, and is associated with inactivation of the tumor suppressor gene *von-Hippel Lindau* (*VHL*) by either genetic and epigenetic factors [76, 77]. *VHL* inactivation in both sporadic and familial forms can occur due to point mutations or deletions at the genetic level, or due to DNA hypermethylation at the gene promoter [76, 77].

DNA methylation also affects the regulation of several genes in RCC and has the potential to be used as biomarker and as a therapeutic target [78]. For example, hypermethylation of *RASSF1A* and *HIC* in patients with RCC is associated with a poor prognosis [79, 80].

Several miRNAs showed altered expression in RCC, resulting in changes to important cellular functions such as apoptosis, angiogenesis and the epithelial mesenchymal transition [27, 81]. Examples of miRNAs with altered expression in RCC include miR-210, miR-34a, miR-30c, miR-29b and miR-23b [27, 81].

### Kidney cancer - current treatment

Radical nephrectomy is the standard of care for localized renal cell carcinoma. However, high rates of recurrence after surgery demand the development of new adjuvant therapies. Both radiotherapy and hormone therapy have proven ineffective in advanced stages of disease and chemotherapy has a response rate inferior to 10% [82, 83].

### Kidney cancer - pre-clinical data

Preclinical studies using epigenetic drugs for kidney cancer treatment show some promise. In renal cancer cell lines, the HDACi Panobinostat induced cell cycle arrest and apoptosis and also resulted in a reduction in tumor size in xenograft mice models [84]. Pre-clinical studies of DNMT inhibitors in kidney cancer have also shown promise with evidence of reactivation of silenced genes and growth inhibition of cancer cells [85, 86].

One ongoing issue in RCC treatment is resistance to immunomodulatory therapy with interferons. This can occur via promoter hypermethylation and silencing of interferon response genes [87]. Treatment of renal cancer cell lines with 5-Aza-2’-deoxycitidine (5-Aza-dC), increased expression of interferon response genes and restored interferon induced apoptosis [87]. In addition, treatment of RCC cells with the antisense DNMT1 oligonucleotide MG98 also restored susceptibility to interferon therapy [88].

Downregulation of miR-30c in RCC is associated with promotion of the epithelial mesenchymal transition [89]. Reduced expression of this miRNA is associated with hypoxia and *VHL* cell status with lower levels of miR-30c being observed in *VHL*-deficient RCC cell lines [89]. Transfection of a RNA mimic to restore miR-30c levels caused an increase in E-cadherin expression and reduced cell migration capacity [89].

### Kidney cancer - clinical trials

Trials of HDACi in RCC have shown mixed responses. HDACi Vorinostat used as a monotherapy showed an objective response in 36% of patients, however 63% of the patients presented disease progression at 6 months (Table 2: s). When used in combination with Bevacizumab, 48, 6 % of the patients showed stable disease at 6 months (Table 2: NCT00324870). By contrast, treatment with Panobinostat alone resulted in no objective responses, and a median of progression-free survival of 1.7 months (Table 2: NCT00550277, [90]). Treatment was generally well tolerated, but 7 patients reported thrombocytopenia grade 3 or higher (Table 2: NCT00550277, [90]). In a separate small phase I trial, the HDACi Entinostat was administered in combination with Isotretinoin. One patient, who had presented with disease progression after treatment with cytokines and anti-angiogenic therapy, subsequently showed stable disease [91]. The patient had a reduction in tumor size after 4 months of therapy and did not show signs of disease progression at 12 months [91]. However, the number of patients enrolled in the study (2) was insufficient to draw any conclusions [91].

**Table 2:** Clinical trials of epigenetic drugs in kidney cancer

Drug	Combined Therapy	Enzimatic Class	Approval Stage	Status	Indication	Results	Reference/Clinical trial identification
Vorinostat	Bevacizumab	HDAC inhibitor	Phase 1/2	completed	Unresectable or metastatic kidney cancer	48,6% of the patients had absence of disease progression at 6 months8,11% of the patients experienced serious adverse events	NCT00324870
Vorinostat	Isotretinoin	HDAC inhibitor	Phase 1/2	Completed	Advanced renal cell carcinoma	MTD of Vorinostat in combination with isotretinoin=0,5 mg/kg	NCT00324740
Vorinostat	-	HDAC inhibitor	Phase 2	Completed	Advanced renal cell carcinoma	An objective response was observed in 36% of the patients63% of the patients demonstrate progressive disease and one patient had serious adverse events	NCT00278395
Vorinostat	Pembrodizumab	HDAC inhibitor	Phase 1	Ongoing	Advanced renal or urothelial cell carcinoma	Final data collection date for primary outcome measure: May 2018	NCT02619253
Panobinostat	-	HDAC inhibitor	Phase 2	Completed	Refractory clear cell renal carcinoma	Median of progression free survival=1,7 months 30% of the patients experienced serious adverse events	Hainsworth *et al*. 2011 (NCT00550277)
Panobinostat	Everolimus	HDAC inhibitor	Phase ½	Terminated	Metastatic or unresectable renal cell cancer	The study has been terminated (patients off study, principal investigator left institute)	NCT01582009
Panobinostat	Sorafenib	HDAC inhibitor	Phase 1	Ongoing	Advanced renal cell carcinoma	Study completion date: November 2016	NCT01005797
Entinostat	Isotreitinoin	HDAC inhibitor	Phase 1	Completed	solid tumor including kidney cancer, urothelial carcinoma and prostate cancer	No objective responses were observed but stable disease was noticed in patients with kidney, prostate and pancreatic cancerRecommended doses for phase 2: 4 mg/m of entinostat once weekly and 1mg/kg of isotretinoin per day	Pili et al. 2012
Entinostat	IL-2	HDAC inhibitor	Phase 1/2	Ongoing	Metastatic kidney cancer	No date given for study completion	NCT01038778
Decitabine	Interferon alpha2B	DNMT inhibitor	Phase 2	Terminated	Advanced renal cell carcinoma	The study was terminated due to low accrual and unavailable treatment agent.	NCT00561912
Decitabine	IL-2	DNMT inhibitor	Phase 1	Completed	Melanoma or renal cell cancer	Three patients with renal cell cancer had stable disease	Gollob *et al*. 2006
GTI-2040	Capecitabine	Antisense oligonucleotide that targets R2 subunit of ribonucleotide reductase	Phase 2	Completed	Advanced/metatastic renal cell carcinoma	52% of the patients had stable disease with median duration of 4 monthsOne partial response was observed	Desai *et al*. 2004
MG98	-	Antisense oligonucleotide that targets DNMT1	Phase 2	Completed	Metastatic renal carcinoma	Six patients had stable disease but no objective responses were seen	Whinquist E. *et al* 2006
Oblimersen	Interferon alpha	Antisense oligonucleotide that targets bcl2	Phase 2	Completed	Metastatic renal cell cancer	No study results or publications provided	NCT00059813
MRX34		RNA mimic	Phase 1	Terminated	Renal cell carcinoma	This study was terminated due to serious adverse events	NCT01829971

The only DNMT inhibitor tested in RCC is Decitabine. When used in combination with the cytokine IL-2 in a phase II study of advanced RCC, three out of five patients showed stable disease [92]. Another study combining Decitabine with interferon-α was terminated early due to low accrual (Table 1, NCT00561912).

Treatment with antisense oligonucleotides has also resulted in stabilization of disease in some RCC patients. The antisense oligonucleotide GTI-2040, targeting the R2 subunit of ribonucleotide reductase, was tested in patients with metastatic disease in a phase II trial and generated a partial response for approximately eight months [93]. One patient experienced a dose limiting toxicity (grade 3 diarrhea) and adverse effects of all grades were reported in this trial including grade 4 pancytopenia, pulmonary embolism and bone pain [93]. In a trial reported by Winquist *et al*, in which the antisense oligonucleotide MG98 was administered to 15 patients, no objective responses were observed with nine patients presenting progression of the disease. MG98 targets DNMT1 but no decrease in enzyme activity was observed [94]. Also, grade 3 and 4 adverse effects forced 8 patients to discontinue treatment, primarily due to elevations in alanine aminotransferase (ALT) and aspartate aminotransferase (AST) levels [94].

miRNA mimics have also been trialed in RCC. MRX34, an miRNA mimic of the tumor suppressor miRNA34, was tested in a phase I clinical trial for advanced or metastatic cancers, including RCC, but the trial was terminated early due to serious immunologic adverse events (Table 2: NCT01829971).

### Bladder cancer - epigenetics

Epigenetic modifications are important in bladder cancer development [95]. An increase in global histone methylation was reported in bladder cancer samples, particularly in the subset of patients with non-muscular invasive bladder cancer. In these patients, a global increase in H3K9 and H3K27 methylation was associated with high-grade tumors. However, the authors did not find any correlation between histone methylation and tumor recurrence or survival [96]. Interestingly, another study by the same group revealed that a decrease in methylation levels of other histone proteins, namely H3K4 and H3K20, could be a prognostic biomarker for muscle invasive bladder cancer [97]. The presence of different histone methylation patterns in muscle invasive and non-invasive bladder cancer, suggests that patients of these subgroups will respond differentially to epigenetic therapies affecting histone methylation. These data reinforce the need for biomarker discovery to predict responses to epigenetic therapy.

With respect to DNA methylation in bladder cancer, Friedrich *et al* reported hypermethylation of the genes *DAPK*, *BCL2* and *TERT* in urine samples from patients with bladder cancer [98]. Detection of methylation patterns in urine samples has proven to be a good diagnostic strategy in bladder cancer. [99, 100]. Methylation of some gene promoters can also be indicative of prognosis, for example, methylation of the *RUNX3* promoter is associated with a higher risk of progression and lower survival [101].

Differential miRNA expression is another epigenetic feature of bladder cancer and can distinguish between cancer patients and healthy subjects [27, 102]. The miRNAs implicated in bladder cancer target genes involved in cell cycle control, cell proliferation, cell differentiation and signal transduction pathways [27, 102]. Both upregulation and downregulation of miRNA expression can potentiate cancer development. In bladder cancer, loss of miR-200 is associated with epithelial mesenchymal transition while upregulation of miR-21 and miR-129 is associated with high grade tumors and poor prognosis, respectively [27, 102, 103].

### Bladder cancer - current treatment

Muscle invasion is a critical factor in the selection of the right therapeutic option for bladder cancer. In the case of non-muscle invasive bladder cancer, the standard clinical approach is transurethelial resection followed by administration of chemotherapeutic or immunotherapeutic agents [104]. For muscle-invasive bladder cancer, a more aggressive form, the standard of care is radical cystectomy [105]. In cases of disease relapse, occurring in approximately 30% of patients, combinatory chemotherapy regimens containing cisplatin are used. These include MVAC (Methrotrexate, Vinblastine, Achiamycin and Cisplatin) and GC (Gemcitabine and Cisplatin). Despite positive early responses to these therapeutic modalities, the median survival rate after treatment is only 12 months [104–106]. For advanced and metastatic bladder cancer, where surgery is not a valid approach, the only treatment option is palliative chemotherapy. This reflects the need for the development and implementation of new therapeutic agents [104].

### Bladder cancer - pre-clinical data

Proteomic studies after exposure of bladder cancer cells to HDAC inhibitors reveals that HDAC activity influences many cellular pathways involved in carcinogenesis [107]. The treatment of bladder cancer cell lines with these agents resulted in cell growth suppression and induction of cell death [107]. Wang *et al* showed that the HDACi Vorinostat was able to induced cell growth inhibition in bladder cancer cells in part due to downregulation of survivin, an apoptosis inhibitory protein [108]. Importantly, Vorinostat had a synergistic effect with chemotherapeutic agents including Cisplatin, Mitomycin c, and Adriamycin. Combined therapy of using Vorinostat and Cisplatin prevented cancer progression in an animal model [108].

Positive responses were also obtained in bladder cancer cells after administration of the DNMTi Belinostat. A significant decrease in cell proliferation was observed *in* vitro, and *in vivo* with the use of a transgenic mouse model [109].

Epigenetic drugs can be used in combination with other agents to enhance their efficacy. Shang *et al* evaluated the DNMT inhibitor 5-Aza-2-deoxycytidine in combination with chemotherapeutic agents in bladder transitional cell carcinoma cell lines. The authors demonstrated that DAC enhances susceptibility to Cisplatin, a common agent used as neoadjuvant therapy for bladder cancer, in a synergistic way. Both agents induced cell cycle arrest at G2/M phase, with Cisplatin also inducing tumor cell apoptosis [110].

Rieger *et al* compared the efficacy and efficiency of siRNAS and antisense oligonucleotides against *Bcl-xL* in bladder cancer cell lines. Their effect in combined therapy with chemotherapeutic agents was also tested. Both agents enhanced tumor cell apoptosis when administrated with Cisplatin, however *Bcl-xL* knockout was more efficient with siRNAs than with antisense oligonucleotides [111]. Another study showed that simultaneous knockout of *Bcl-xL* and *survivin* with siRNAs in bladder cancer cells led to greater sensitization of the cells to chemotherapeutic agents [112].

### Bladder cancer - clinical data

Relative few studies of epigenetic therapies have been undertaken in bladder cancer. Only one of three clinical trials employing HDAC inhibitors showed a positive response; in this trial, four of fifteen patients treated with the HDACi Belinostat in combination with Carboplatin or Paclitaxel showed complete or partial response to treatment, while five patients presented disease progression with a median time to progression of 136 days (Table 3, NCT00421889). Further studies employing this HDACi in combination with other agents are essential to confirm its therapeutic potential [109]. The only study using the HDACi Vorinostat terminated due to lack of efficacy (Table 3, NCT00363883). Three other phase 2 clinical trials employing epigenetic drugs for bladder cancer treatment are currently ongoing (Table 3: NCT02236195, NCT00978250, NCT01780545).

**Table 3:** Clinical trials of epigenetic drugs in bladder cancer

Drug	Combined Therapy	Enzimatic Class	Approval Stage	Status	Indication	Results	Reference/Clinical trial identification
Belinostat	Carboplatin or paclitaxel	HDAC inhibitor	Phase 1/2	Phase 1 concluded, Phase 2 ongoing	Bladder cancer	Four out of fifteen patients had complete or partial response patients had progressive disease with median time to progression of 136 days	NCT00421889
Vorinostat	-	HDAC inhibitor	Phase 2	Terminated for futility	Locally Recurrent or Metastatic Cancer of the Urothelium	No objective response was observedMedian overall survival: 4,3 monthsMedian progression free survival: 1,1 months	NCT00363883
Mocetinostat	-	HDAC inhibitor	Phase 2	Ongoing	Patients with advanced urothelial Carcinoma and inactivating alterations of acetyltransferase genes	Study completion date: December 2017	NCT02236195
FdCyd	Tetrahydrouridine	DNMT inhibitor	Phase 2	Ongoing	Advanced cancer including bladder Cancer	Study completion date: May 2017	NCT00978250
OGX-427	Docetaxel	Antisense oligonucleotide that targets heat shock protein 27	Phase 2	Ongoing	Advanced urothelial Carcinoma	Study completion date: February 2017	NCT01780545

## CONCLUSIONS

Despite the obvious importance of epigenetics in the development of cancer, few epigenetic therapies have thus far reached advanced clinical testing. As the data above demonstrates, pre-clinical data has not translated into the hoped-for clinical responses. This is likely secondary to the nonspecific actions of epigenetic drugs and the consequent toxicities associated with their administration.

Many of the epigenetic therapies being tested have global epigenetic effects on both cancerous and non-cancerous tissues. Moreover, some of them have additional non-epigenetic effects that limit their efficacy. HDAC enzymes, for instance, target non-histone proteins involved in oncologic pathways unrelated to epigenetic regulation [113]. It is important to consider that the observed therapeutic responses to HDACi treatment may thus be the result of the altered activity of these proteins and not to the reversal of specific epigenetic marks [113]. Similarly, demethylating agents are not specific to genes involved in carcinogenesis but result in global demethylation of the genome, an epigenetic signature associated with genomic instability that can lead to severe side effects [113].

Of all the clinical trials analyzed in this study, 16 included evaluation of gene expression and/or DNA methylation as a secondary objective of the trial. We feel that analysis of gene expression and epigenetic patterns should be included in all clinical trials using epigenetic agents in order to assess the causal link between drug induced alterations and therapeutic responses. A better knowledge of the specific mechanism of action of these agents is essential to overcoming their clinical limitations and improving therapeutic success.

Antisense oligonucleotides (ASOs) avoid some of the issues described above as as they are designed to be more target specific. Thus far, two separate studies of ASOs as monotherapy have shown no objective responses [71, 94], however when used in combination with other agents the results have been more promising [66, 93]. However, ASOs also have their limitations secondary to toxicity and delivery efficiency [67, 69].

Currently, cancer treatment is determined largely according to cancer stage, even though patients with similar stage cancers may respond differently to the same type of therapy. The promise of “personalized medicine” is the idea that tailoring treatment to an individual patient will optimize efficacy while minimizing toxicity. Personalization should be an ongoing goal for all cancer therapy development, including epigenetic therapies. Biomarker development should thus be a central goal in the development of epigenetic therapies, both so that the correct patient receives the correct therapy, and to ensure that therapies that have value in a subset of patients are not passed over because of lack of efficacy in other patients. Epigenetic therapies are still in their infancy as a therapeutic class and pre-clinical promise has not yet translated into clinical efficacy. However, the development of target-specific agents, and the careful combination of epigenetic therapies with traditional modalities should enable them to achieve clinical success in the near future.
